# Behavior of Calcium Phosphate–Chitosan–Collagen Composite Coating on AISI 304 for Orthopedic Applications

**DOI:** 10.3390/polym14235108

**Published:** 2022-11-24

**Authors:** Claudio Zanca, Bernardo Patella, Elisa Capuana, Francesco Lopresti, Valerio Brucato, Francesco Carfì Pavia, Vincenzo La Carrubba, Rosalinda Inguanta

**Affiliations:** 1 Department of Engineering, University of Palermo, Viale delle Scienze, 90133 Palermo, Italy; 2 Consorzio Universitario di Caltanissetta, Corso Vittorio Emanuele 92, 93100 Caltanissetta, Italy; 3 ATeN Center, University of Palermo, Viale delle Scienze, 90133 Palermo, Italy

**Keywords:** coating, corrosion, galvanic deposition, hydroxyapatite, chitosan, collagen, AISI 304, cytotoxicity

## Abstract

Calcium phosphate/chitosan/collagen composite coating on AISI 304 stainless steel was investigated. Coatings were realized by galvanic coupling that occurs without an external power supply because it begins with the coupling between two metals with different standard electrochemical potentials. The process consists of the co-deposition of the three components with the calcium phosphate crystals incorporated into the polymeric composite of chitosan and collagen. Physical-chemical characterizations of the samples were executed to evaluate morphology and chemical composition. Morphological analyses have shown that the surface of the stainless steel is covered by the deposit, which has a very rough surface. XRD, Raman, and FTIR characterizations highlighted the presence of both calcium phosphate compounds and polymers. The coatings undergo a profound variation after aging in simulated body fluid, both in terms of composition and structure. The tests, carried out in simulated body fluid to scrutinize the corrosion resistance, have shown the protective behavior of the coating. In particular, the corrosion potential moved toward higher values with respect to uncoated steel, while the corrosion current density decreased. This good behavior was further confirmed by the very low quantification of the metal ions (practically absent) released in simulated body fluid during aging. Cytotoxicity tests using a pre-osteoblasts MC3T3-E1 cell line were also performed that attest the biocompatibility of the coating.

## 1. Introduction

Despite the technological development and continuous research on materials with high performance, metallic materials always remain the best choice to fabricate orthopedic devices such as screws, pins, dentures, or dental implants [1,2]. Nevertheless, the surface of a metallic implant does not have good osteointegration [3,4]. The modification of metallic surfaces represents one of the most used techniques employed to improve the interaction between human bones and orthopedic implants. Differing from physical treatments that aim to increase surface roughness and ensure good adhesion and cellular differentiation [5,6], the realization of biomimetic coatings could be a viable solution because they improve the interaction with periprosthetic tissues.

In this work, the attention was focused on a composite coating of calcium phosphates (CaP), chitosan (CS), and collagen (CL), taking inspiration from the hierarchical structure of bone tissue [7,8]. Bone tissue consists of bone cells and an extracellular matrix that is constituted of organic and inorganic components. The organic portion of tissue consists of 90% CL and other proteins such as osteocalcin, osteonectin, and osteopontin that confer tensile strength to the bone and support the mineralized matrix [9]. The latter contains mainly Ca and P in the form of hydroxyapatite crystals that confer mechanical strength, but also, other numerous elements are present [10,11]. Hydroxyapatite (HA, Ca_10_(PO_4_)_6_(OH)_2_) is a ceramic calcium phosphate compound largely used in the orthopedic field to obtain coatings and scaffolds [12]. In addition, HA is already employed in the biomedical field due to its great biocompatibility and osteoconductivity, which increase the strong connection between bone and implanted devices [13,14]. According to Kim et al., HA coatings via sol-gel deposition enhance osteoblastic activity in vitro because of their excellent crystallinity and roughness, which gives a good response with bone cells [15]. In addition to biocompatibility, it is also important to consider that the presence of a well-adhered and compact coating could act as a barrier between the metal and periprosthetic tissues [16,17,18]. As soon as the implant is installed inside the body, corrosion phenomena can occur on the metal surface due to the action of aggressive species such as chlorides richly present in body fluids. The occurrence of corrosion phenomena causes the release of metal ions or nanoparticles that can produce adverse local tissue reactions around periprosthetic tissues during the post-surgery period [19].

In this context, the use of biopolymers can give added value in terms of performance, and in particular, CS has been extensively adopted for composite coatings [20,21,22,23]. CS is a linear polysaccharide compound, and it was synthesized for the first time by C. Rouget in 1859 through the alkaline deacetylation of chitin, a homopolymer of β-1,4-N-acetyl-D-glucosamine. Chitin is extracted predominantly from crab and shrimp exoskeletons and fungi [24,25,26]. The biological activity of CS represents a crucial factor associated with deacetylation degree, and it is directly related to the amount of amino groups on the hydrocarbon backbone of CS [27,28]. This biopolymer solves several technological solutions such as in food and beverage technology, cosmetics, agriculture, water waste treatment, and pharmaceutics [29,30]. In the last decades, numerous applications of CS have had beneficial repercussions in biomedical fields because of its biocompatibility, biodegradability, and antimicrobial and mucoadhesive properties. Recent studies were carried out in bone healing, wound healing, drug delivery, tissue engineering, and biosensors [31,32,33].

As mentioned before, CL is the most plentiful protein in bone tissue. It promotes the adhesion and proliferation of osteoblasts and improves the biocompatibility of the coating [34,35]. This protein was employed in several studies regarding composite coating on biodegradable metals [36,37], titanium alloys [38,39,40], and stainless steel [41,42]. In addition, CL not only has angiogenic promotive properties [43,44] but, also, CL fibrils might be able to chelate calcium ions in order to work as a nucleation site for CaP, stimulating the development of a coating close to the natural bone [45].

The coating proposed in this work is aimed to inhibit corrosion phenomena and, exploiting the characteristics and peculiarities of the three components, increase the biocompatibility of the orthopedic prosthesis to extend its lifetime inside the human body. Another aspect to highlight is the deposition method adopted to coat the metal substrate. In particular, galvanic deposition was used because it is able to realize biocompatible coating [46,47,48,49,50,51]. The distinctive feature of this technique is that it does not require any external power supply. The galvanic contact between the working electrode and the sacrificial anode drives the whole process in an electrochemical cell. In this process, the difference in the electrochemical redox potential of the galvanic couple plays a crucial role in depositing coatings on the metallic substrate [52]. Additionally, galvanic deposition is a scalable and controllable process because it is based on the ratio of exposed areas between the anode and cathode. This consolidated and very versatile technology is also appropriate for producing numerous materials [53,54,55] in the nanostructured form [53,54,55,56,57,58,59,60,61,62,63,64]. In our other previous works, CaP-based, CS-based, and composite coatings were obtained through galvanic deposition on stainless steel [65,66,67,68,69]. In these works, we have demonstrated that, among other things, galvanic deposition is also able to produce coatings with good adhesion on the substrates and a lack of cytotoxicity.

In our previous work, the preliminary results on the fabrication of a calcium phosphate–chitosan–collagen composite coating on AISI 304 were reported [70]. Here, the behavior of these coatings was studied in detail. Corrosion tests were carried out in simulated body fluid (SBF) emulating the human body environment. Physical-chemical characterizations of the coatings were carried out to investigate the morphology and chemical composition. Furthermore, the release of metal ions from the coating in SBF was studied. The results show that the obtained composite coatings can slow down the corrosive processes, and also, they do not produce any kind of cytotoxic problems.

## 2. Materials and Methods

### 2.1. Materials

The composite coatings were fabricated on AISI 304 (UNS S30400, 0.025% wt. C, 18.18% wt. Cr, 8.03% wt. Ni, 1.66% wt. Mn, 0.31% wt. Si, 0.031% wt. P, and 0.001% wt. S, and Fe at balance) in the form of bars (1.5 cm × 7 cm × 0.3 cm). Zn (sheets of 3 cm × 7 cm × 0.1 cm) was used as a sacrificial anode. Prior to carrying out galvanic deposition, the electrodes were mechanically pretreated. Initially, the metallic surfaces were degreased in an ultrasonic bath in pure acetone for 10 min. Afterward, mechanical polishing with abrasive papers (#150, #300, #800, #1200) was carried out. Finally, an ultrasonic washing was conducted in deionized water and acetone three times, each lasting 5 min. After the degreasing step, the surface was delimited with an insulator lacquer to expose an active area of 1.13 cm^2^ and 27 cm^2^ for the cathode and anode, respectively. The cathodic solution was obtained using calcium nitrate tetrahydrate (0.061 M), ammonium dihydrogen phosphate (0.036 M), sodium nitrate (0.1 M), and lactic acid (0.08 M). This solution was prepared at 40 °C and under continuous stirring. After the solubilization of the all salts, 5 gL^−1^ of CS and 0.2 gL^−1^ of collagen (type I) were added. The anodic solution consisted of sodium chloride (1 M).

### 2.2. Galvanic Deposition

Galvanic deposition was carried out in a two-compartment electrochemical cell connected via a saturated potassium chloride salt bridge. A scheme of the apparatus can be found in our previous works [67,68,69,71]. The electrodes were short-circuited through a copper wire. A fresh solution was used for each experiment. The galvanic deposition process was performed at 50 °C for 24 h by using a heating chamber with natural convection and an uncontrolled internal atmosphere (Binder, mod ED56, Tuttlingen, Germany). After deposition, the samples were washed with distilled water and left to air dry before characterizations.

### 2.3. Morphology Analysis

The morphology of the coatings was examined using an FEG-ESEM microscope (model: QUANTA 200, FEI, Hillsboro, OR, USA) equipped with an energy dispersive spectroscopy (EDS) probe. EDS was performed in different areas of the sample to investigate its homogeneity. In the text, the average values of deposit composition were reported.

### 2.4. X-ray Diffraction Analysis

The crystallographic structures were studied by X-ray diffraction using a RIGAKU instrument (model: D-MAX 25600 HK, Tokyo, Japan). The analyses were carried out in the 2-theta range from 10° to 60° by means of copper Kα radiation (λ = 1.54 Å, setup conditions: tube voltage 40 kV, current 30 mA, scan speed 4°min^−1^, sampling 0.01°). The results of X-ray diffraction were studied and compared with the ICDD database [72].

### 2.5. Raman Spectroscopy and FT-IR/ATR Analysis

The Raman spectra were obtained using a Renishaw (model: inVia Raman Microscope, Wotton-under-Edge, UK) spectrometer. The excitation was provided by the 532 nm line of a Nd:YAG laser calibrated by the Raman peak of polycrystalline Si (520 cm^−1^). The Raman spectra were analyzed via comparison with the RHUFF database. FT-IR/ATR analyses were carried out by using an FT-IR/NIR Spectrum 400 spectrophotometer (Perkin-Elmer Inc., Wellesley, MA, USA). The spectra were collected in the range of 4000–500 cm^−1^.

### 2.6. ICP-OES Analysis

To quantify the metal ion concentration released from the sample after 21 days of aging in SBF at 37 ± 1 °C, inductively coupled plasma optical emission spectrometry (ICP-OES, PerkinElmer Optima 2100 DV, Waltham, MA, USA) was also executed. In particular, the concentration of Fe, Ni, Cr, Ca, and P was quantified by ICP-OES. Prior to the sample analysis, for each element, a calibration line was obtained using standard calibration solutions.

### 2.7. Corrosion Tests

The behavior of the coating against corrosion phenomena was studied by immersing the samples in an SBF solution, prepared according to the procedure reported in [66], for a period of 21 days at a temperature of 37 °C. SBF was prepared using MilliQ water (18 MΩcm) produced with an AQUA Max system (JOUNGLIN, Basic 360 and Ultra 370, Gyeonggi-do, Korea). MilliQ water was heated at 37 ± 1 °C under stirring at 200 rpm. Then, the salts were added in the same order reported in Table S1 (in Supplementary Materials). Separately, 11.93 g of HEPES (2-(4-(2-hydroxyethyl)-1-piperazinyl)-ethanesulfonic acid) was added in 100 mL of MilliQ water at 37 °C, which afterward was mixed into the first solution. pH was kept up to 7.40 with 0.8 mL of NaOH 1.0 M. The corrosion test consisted of the monitoring of (OCP), potentiodynamic polarization (PP), and electrochemical impedance spectroscopy (EIS) in a conventional three-electrode cell with a Pt wire as the counter electrode and a 3.0 M Ag/AgCl as the reference electrode [65,66,67,68,69]. Corrosion potential (E_corr_) and corrosion current density (i_corr_) were calculated by extrapolation of Tafel’s curves. The polarization measurements were performed with a scan rate of 0.166 mVs^−1^ in a potential range of ±150 mV with respect to the OCP value. EIS was carried out in the range frequency from 100 kHz to 0.1 Hz with 0.010 V of AC perturbation. The impedance data were fitted using ZSimpWin software (Ametek, Berwyn, PA, USA). using an equivalent circuit (EC).

### 2.8. Cytotoxicity

For cytotoxicity tests, the samples (1.5 cm× 3 cm × 0.3 cm) were first sterilized through soaking in a 70% ethanol bath for 24 h with UV light exposure. Each sample was then incubated with Dulbecco’s Modified Eagle Medium (DMEM, Sigma Aldrich, St. Louis, MO, USA) at 37 °C for 24 h at a volume-to-surface area ratio of 5 mLcm^−2^. Subsequently, the treated media were collected in a 50 mL Falcon to carry out cytotoxicity analyses. MC3T3-E1 Pre-osteoblastic cells, purchased from Sigma-Aldrich (ECACC), were cultured in DMEM supplemented with 10% fetal bovine serum, 1% glutamine, and 1% antibiotic at 37 °C and in a 5% CO_2_ atmosphere. Cells were seeded into wells of a 24-well culture plate at the concentration of 3 × 10^3^ cells/well and incubated with normal DMEM at 37 °C and 5% CO_2_. After 24 h, the medium was replaced with the treated medium. Cytotoxicity tests were conducted after 0, 1, 5, and 8 days of culture. Cell viability was assessed with AlamarBlue cell viability reagent (Invitrogen, Waltham, MA, USA). Each well was incubated for 3 h with 500 μL of AlamarBlue reagent (10×) diluted (1:10) in DMEM. The resulting fluorescence was read on a plate reader at an excitation wavelength of 530/25 (peak excitation is 570 nm) and an emission wavelength of 590/35 (peak of emission is 585 nm). Each experiment reported in this work was repeated at least three times.

The determination of the cell number was carried out with a standard curve, prepared seeding a known number of cells (from 10^3^ up to 2 × 10^5^) into wells. After two hours, wells were incubated at 37 °C with AlamarBlue following the procedure described above. The calibration curve was obtained by plotting the number of the seeded cells as a function of the read fluorescence.

## 3. Results

### 3.1. Galvanic Deposition

Immediately after the electrodes were short-circuited and immersed in their solutions, at both electrodes, a series of reactions occurred. Specifically, at the anodic compartment, the dissolution of zinc occurred according to the following reaction (1):

Zn → Zn^2+^ + 2e^−^   (E° = −0.76 V/NHE) (1)

Electrons generated due to this anodic reaction moved to the working electrode, which operated as the cathode, where the reduction reactions took place, in particular, the reactions of electro generation of base produce hydroxyl ions on the surface of the cathode. Nitrate ions, water molecules, and dissolved oxygen in solution were involved according to the reactions (2)–(4) [73,74,75,76,77,78]:

NO_3_^−^ + H_2_O + 2e^−^ → NO_2_^−^ + 2OH^−^   (E_eq_ = 0.0835 − 0.059 pH V/NHE) (2)

2H_2_O + 2e^−^ → 2OH^−^ + H_2_              (E_eq_ = 0.0 − 0.059 pH V/NHE) (3)

O_2_ + 2H_2_O +4e^−^ → 4OH^−^                 (E_eq_ = 1.23 − 0.059 pH V/NHE) (4)

Since the formation of the composite coating consists of a co-deposition, the reactions that lead to the deposition of calcium phosphate and biopolymers must occur simultaneously. With regards to calcium phosphate, the mechanism of deposition occurs due to equilibrium reactions related to the phosphate ions dissociation. In particular, the increase in pH at the electrode interface due to the electrogeneration base reactions shifts the dissociation equilibrium of H_2_PO_4_^−^ toward HPO_4_^2−^ (5):

H_2_PO_4_^−^ + OH^−^ ↔ HPO_4_^2−^ + H_2_O   (pH = 7.19) (5)

The formation of hydrogen phosphate ions leads to the precipitation brushite (BS) (CaHPO_4_·2H_2_O) according to the reaction (6):

Ca^2+^ + HPO_4_^2−^ + 2H_2_O → CaHPO_4_·2H_2_O (6)

BS is an electrically insulating compound. Therefore, if a compact and uniform coating is deposited, the electrode surface is no longer exposed to the solution electrolyte, and consequently, the hydroxyl ions would not be produced at the interface. However, in our case, a non-uniform and porous layer of BS was formed and the electrogeneration of base reactions was not hindered. Consequently, HPO_4_^2−^ ion continued to dissociate, forming the orthophosphate ion according to the equilibrium (7):

HPO_4_ ^2−^ + OH^−^ ↔ PO_4_^3−^ + H_2_O   (pH = 12.03) (7)

As soon as pH reaches a value above 12, HA precipitation occurs following the reaction (8):

10Ca^2+^ + 6PO_4_^3−^ + 2OH^−^ → Ca_10_(PO_4_)_6_(OH)_2_ ↓   (pH > 12) (8)

Regarding biopolymers, the addition of lactic acid during the preparation of the cathodic solution causes the solubilization of the polymeric chains. This is due to the protonation of the amine groups present in the hydrocarbon backbone. As per the mechanism of chitosan deposition, it is also due to the pH increase at the electrode/electrolyte interface. This increase leads to the precipitation of the polymer according to the reaction (9):

Chit-NH^3+^ + OH^−^ → Chit-NH_2_ + H_2_O   (pKa 6.2–6.4) (9)

Collagen is characterized by an isoelectric point around a pH of 7.4. According to a study of Ling et al. the pH gradient interface plays a key role in composite formation at the electrode/electrolyte [79]. Basically, the increase in pH causes the generation of calcium phosphate crystals, and simultaneously, collagen fibrils assemble and mineralize near the cathode surface. Although the increase in pH causes a negative charge on the carboxyl groups, as a matter of fact, carboxyl groups act as nucleation points for the calcium phosphate crystals [80,81]. In the meantime, collagen fibers are incorporated within the coating. Therefore, this mechanism allows us to obtain a composite structure. According to Wang et al., the presence of chitosan might contribute to changing the isoelectric point of collagen since interactions are established between the biopolymer chains [82]. In a more recent study, the same mechanism was proposed for the formation of the composite between calcium phosphates and proteins, collagen/BSA, by electrochemical deposition [83].

### 3.2. Morphological Analysis

**Figure 1:**
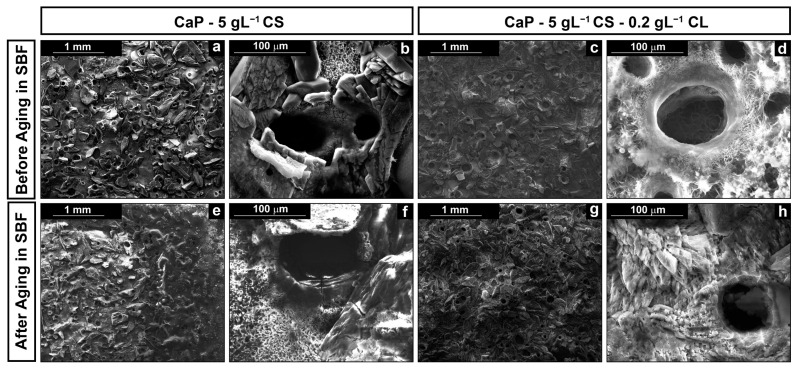
SEM images of the surface of the coatings at different magnifications: (**a**,**b**) CaP/CS and (**c**,**d**) CaP/CS/CL before aging; (**e**,**f**) CaP/CS and (**g**,**h**) CaP/CS/CL after aging in SBF.

The SEM images of the composite coating with and without collagen and before and after the aging in SBF are reported in Figure 1.

SEM images reveal that galvanic deposition allows us to deposit the coating on the entire metallic surface exposed to the cathodic solution. In Figure 1a–d, a massive deposition of the CaP crystals can be observed after deposition. However, the presence of biopolymers was not detected since co-deposition creates an intimate structure between the CaP crystals and polymeric macromolecules. The addition of CL does not contribute to a substantial modification of the structure. It can be interesting to highlight the presence of circular macropores. This peculiarity is attributable to the formation of chitosan in synergy with the hydrogen evolution reaction (HER) [46,84,85] during the deposition. Specifically, the final effect is a porous coating since the bubbles act as a dynamic template [86,87]. According to Mąkiewicz et al. [88], the high viscosity of the solution promotes the adhesion of hydrogen bubbles on the cathode surface during deposition. This phenomenon creates a barrier at the interface electrode/electrolyte that limits the deposition of the coating in that area. Nevertheless, as soon as the bubble reaches a critical size value, the bubbles detachment occurs, and therefore, the deposition begins again in the active area previously occupied. In fact, although the holes appear hollow, their surface is covered by a thinly deposited layer, as shown in the inset of Figure 1b.

**Figure 2:**
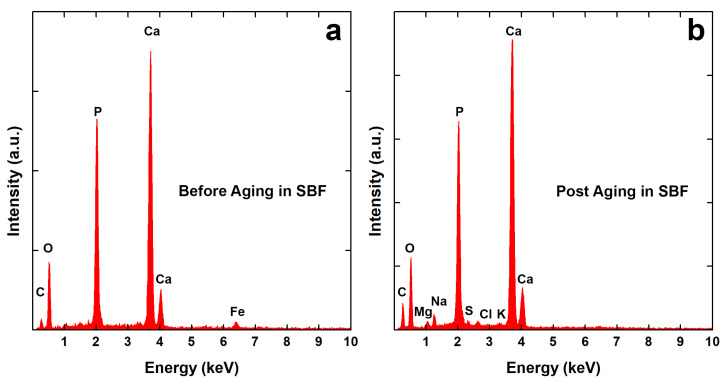
EDS spectra of CaP/CS/CL coating: (**a**) before and (**b**) after aging in SBF.

Coating morphology was also characterized after 21 days of aging in SBF. In previous studies [66,68], we have observed that this time is sufficient to achieve a stable behavior in the calcium-phosphate-based coatings. In both cases, the deposit covered the metallic substrates, as shown in Figure 1e–h, with almost the same morphological characteristics described above. From a chemical composition point of view, EDS data give interesting semi-qualitative information, as shown in Figure 2.

In Table 1, Ca/P and Ca/Fe atomic ratios were reported. The first one is useful to evaluate the coating composition, while the second ratio offers qualitative information concerning its thickness. The data reported in Table 1 indicate that coatings are stable and they are constituted by a mixture of BS (Ca/P = 1) and HA (Ca/P = 1.59~1.86) according to the literature [89]. Fe atoms, coming from the steel substrate, were detected only in the EDS spectra of the as-prepared coatings, Figure 2a. Even if a BS phase was present in the as-deposited coating, after 21 days of aging, a total conversion into HA was observed. Furthermore, Figure 2b shows the presence of additional atoms (Cl, K, Na, Mg) due to chloride salts or the formation of other types of substances that are incorporated within the coating [66,68]. Kumar et al. [90] have demonstrated the transformation of BS coating into HA during aging in SBF. In particular, they have shown that a continuous dynamic process of dissolution/reprecipitation occurs. This is in agreement with the mechanism proposed by Nur et al. [91]. According to these authors, a reversible equilibrium is established between the BS and HA phases in SBF according to the reaction (10):

10CaHPO_4_ + 2OH^−^ ↔ Ca_10_(PO_4_)_6_(OH)_2_ + 4PO_4_^3−^ + 10H^+^ (pH < 12) (10)

The disappearance of Fe in the coating after aging suggests that there is a change in the coating thickness. This is due to the continuous coating dissolution/precipitation process that occurs in SBF, leading, as reported in the literature, to the increases in the coating thickness. In particular, new crystals of calcium phosphate coming from the SBF are formed and are incorporated along with other elements, as discussed above, into the coating [92]. The increase in thickness is also confirmed by XRD patterns where, after aging, the diffraction peaks of the substrate are practically not present.

**Figure 3:**
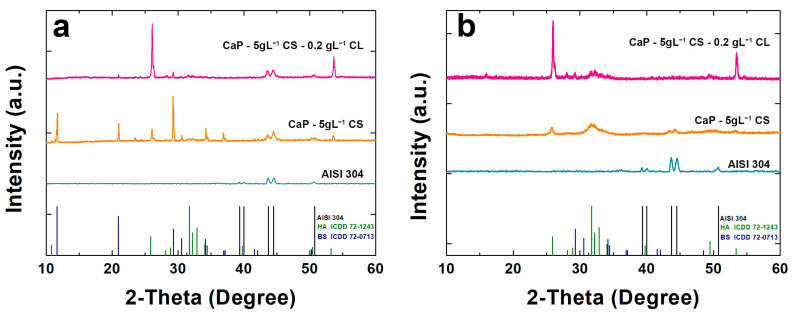
X-ray diffraction patterns of CaP/CS and CaP/CS/CL coatings: (**a**) before and (**b**) after aging in SBF. The pattern of uncoated AISI 304 was also reported for comparison.

Figure 3 shows the XRD patterns of the coated samples. In addition, XRD analysis was carried out also on AISI 304 to emphasize the shielding of peaks due to the presence of coatings. In Figure 3a, BS peaks were identified for 2-theta equal to 11.65°, 20.95°, 29.29°, and 30.54°. Furthermore, a peak for 2-theta equal to 25.87° relative to HA was observed. Unfortunately, it was not possible to attest to the presence of biopolymers in the composite coating. Nevertheless, an increase in the HA peaks was observed in the CaP/CS/CL coating where the main peak of HA (2-theta = 25.87°) was more intense with respect to the CaP/CS coating. After aging, the peaks of BS disappeared (Figure 3b), while new HA peaks emerged for 2-theta equal to 25.87°, 31.74°, 32.18°, 32.87°, and 34.045°. These results are in line with the equilibrium between CaP compounds described above [90,91].

### 3.3. Raman Spectroscopy and FT-IR/ATR Analysis

**Figure 4:**
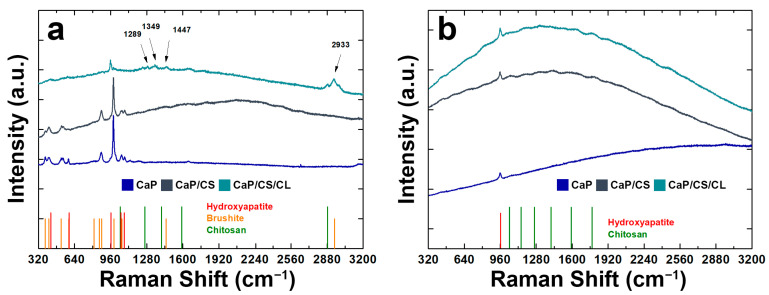
RAMAN spectra of CaP/CS and CaP/CS/CL coatings: (**a**) before and (**b**) after aging in SBF. The spectrum of the CaP coating was also reported for comparison.

Raman spectra were shown in Figure 4. For comparison with the composite coating, Raman analysis was also performed on the CaPs sample without polymers. Through this comparison, it was possible to see the effect of the presence of the polymeric matrix within the coating structure [93,94,95]. BS is characterized by high splitting, and band shifts can be attributed to the protonated phosphate group. In particular, the stretching of the phosphate groups (ν_1_ P-O) can be observed at 985 cm^−1^ and 878 cm^−1^. The Raman modes at 1081 cm^−1^ and 1059 cm^−1^ were related to the stretching of the group PO_4_ (ν_3_ P-O). The vibrational modes at 379 cm^−1^ and 415 cm^−1^ refer to the bending of the HPO_4_ group (ν_2_ O-P-O). A less intense band was observed at 1121 cm^−1^, which can be attributed to the stretching of the HPO_4_^2−^ group (ν_3_ P-O). In addition, the bands related to the bending of the PO_4_ (ν_4_ P-O: 593 cm^−1^) and to the stretching of the HPO_4_^2−^ group (ν_4_ O-P-O: 530 cm^−1^) were observed. In CaP/CS/CL coating, the typical HA stretching mode (ν_1_: 960 cm^−1^) is more intense with respect to the BS ones [94,96,97]. Signals related to chitosan can be traced in the range between 1000~1500 cm^−1^ and are due to stretching of the -CH_2_- groups of the polymer [98,99,100]. The presence of collagen was confirmed by RAMAN modes at 1289 cm^−1^ for Amide III, at 1349 cm^−1^ related to bending (δCH), 1447 cm^−1^ and 2933 cm^−1^ related to the deformation of -CH_2_- and -CH_3_, respectively [101,102]. After 21 days of aging in SBF, the typical HA vibrational mode was noted at 960 cm^−1^ as the only crystalline phase (Figure 4), in agreement with the XRD patterns previously shown. Typical fluorescence interference, due to the polymer matrix of the coating, was also observed [103]. The absence of collagen peaks is due to the mineralization of collagen during aging in SBF [104,105,106].

**Figure 5:**
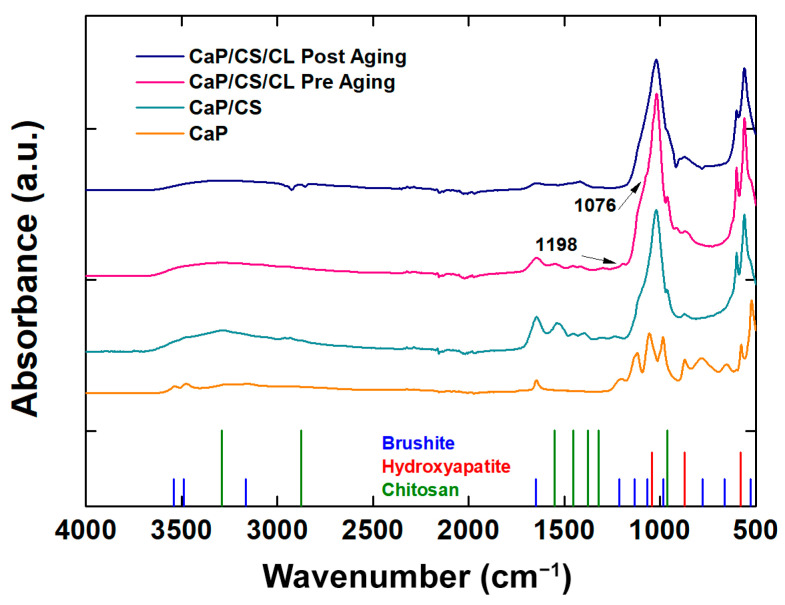
FT-IT/ATR spectra of CaP/CS and CaP/CS/CL coatings before and after aging in SBF. The spectrum of the CaP coating was inserted for comparison.

For further confirmation of the presence of biopolymers within the composite, FT-IR/ATR analyses were performed (Figure 5).

The addition of chitosan revealed main absorption bands in the range 1700–1000 cm^−1^ such as amine I C=O stretching (1627 cm^−1^) and NH bending (1071 cm^−1^). In the range 3600–3000 cm^−1^, the typical broad band related to the absorption of the -OH stretching of the hydroxyl group, where the polymer matrix is present, is present [98,107]. The peaks related to the presence of collagen are the stretching ν(C-OC) at 1076 cm^−1^ and the bending δ(N-H) at 1198 cm^−1^ [108]. With regard to the inorganic component of the coating, a definite absorption peak was observed in all samples for 1648 cm^−1^ related to the bending of the H-O-H bond. The presence of the HA phase in the composites is confirmed by the very intense peak at 1040 cm^−1^ belonging to the asymmetric stretching of the phosphate group. Both the BS and HA phases are present in both coatings in agreement with the results obtained by XRD and Raman [109,110,111,112]. The spectrum of the aged CaP/CS/CL sample in SBF solution at 37 °C for 3 weeks was also collected to analyze its effect on the mineral phase of the coating. By comparing CaP/CS/CL spectra before and after aging, the main changes can be related to a slight decrease in the typical brushite peaks associated with the stretching frequencies (νOH: 3540–3153 cm^−1^) and bend mode (δOH: 1642 cm^−1^). Furthermore, an increase in the peaks attributable to the HA phase, in particular at 870 cm^−1^ (νCO_3_^2−^), can be observed [113]. This result is consistent with that obtained in our previous work [68].

### 3.4. Corrosion Tests

**Figure 6:**
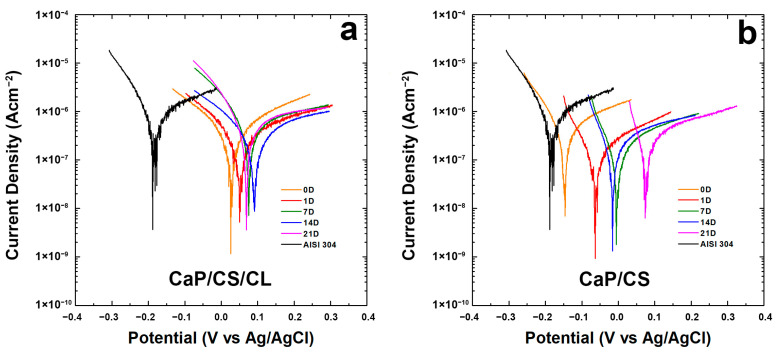
Tafel plots of the coating during 3-weeks of aging in the SBF solution: (**a**) CaP/CS/CL and (**b**) CaP/CS. Tafel curves were recorded immediately after immersion (0 D), after 1 (1 D), 7 (7 D), 14 (14 D), and 21 (21 D) days of immersion in SBF. The Tafel curve of the bare steel was also reported for comparison.

To scrutinize the protective action of the coating, electrochemical tests were performed in vitro using SBF solution at 37 °C. Each corrosion test involved a first step in which the monitoring of OCP was executed for 30 min. This operation is necessary not only for stabilizing the system but also because the presence of an abrupt spike of potential could be a symptom of a chemical instability in the coating in SBF. In Figure S1 (in Supplementary Materials) the results of the OCP measurements were reported. The OCP of uncoated AISI 304 was also added for comparison. Considering the entire period of aging, it can be possible to highlight that the composite coatings hold higher OCP values than bare steel. Thus, the deposit on the metallic surface ensures a barrier effect. Although irregular and porous morphologies were found in Figure 1, the protective skills of the coating were not affected. In fact, the OCP value remains almost constant, with a minor variation of no more than 10 mV during 30 min. Further confirmation comes from the polarization curves reported in Figure 6a,b. In Table 2, values of E_corr_ and i_corr_ values were calculated by fitting of Tafel’s curves.

In particular, in both coatings, a higher potential was observed during the aging compared to the uncoated steel. As per CaP/CS/CL shown in Figure 6a, all the curves remain higher than uncoated steel and shift toward nobler potentials. The same trend was noticed for both samples, even if the CaP/CS/CL sample revealed more positive values of E_corr_ with respect to CaP/CS (Figure 6b). In Figure 6a, it can be observed that from the beginning of aging in SBF, polarization curves move within the range of 0–100 mV and the E_corr_ values remain positive and higher than bare steel [114]. A slight decrease in E_corr_ was observed on the 21st day of aging. In agreement with the literature data, these fluctuations in i_corr_ value are an additional proof of the continuous evolution of the coating in SBF where BS/HA equilibrium was established [115,116]. The consequence of this dynamic development is also the change in coating thickness, as discussed above, leading to the different corrosion resistance during aging in SBF. For CaP/CS, in Figure 6b, at the end of the 21st day of aging, an increase of approximately 200 mV from the beginning with a slight decrease in i_corr_ was attested. From these data, it is possible to conclude that the composite coating CaP/CS/CL is more stable than CaP/CS. It is important to underline, the coatings cannot hinder phenomena corrosion, but they are able to decrease the rate of metal dissolution inside the human body.

**Figure 7:**
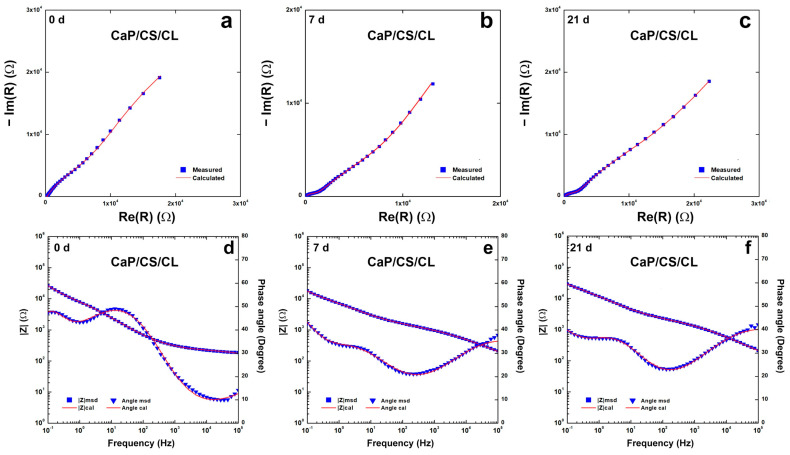
Impedance spectra of CaP/CS/CL coating during 3-weeks of aging in SBF solution: (**a**–**c**) Nyquist plots; (**d**–**f**) Bode plots. Impedance spectra were recorded immediately after immersion (0 D), after 1 (1 D), 7 (7 D), 14 (14 D), and 21 (21 D) days of immersion in SBF.

To evaluate the protective characteristics of the coating, EIS characterizations were performed on CaP/CS/CL samples and Bode and Nyquist plots are reported in Figure 7. Impedance data were fitted using an equivalent circuit R_s_(CPE_1_R_1_)(CPE_2_(R_2_(CPE_3_R_3_))), shown in Figure S2 (in Supplementary Materials). This model was proposed by Orazem and Tribollet [117], and the values are reported in Table S2 (in Supplementary Materials). R_s_ is related to the resistance of the solution. CPE_1_ and R_1_ are related to the outer layer of the coating in contact with the solution. CPE_2_ was inserted to model capacitance of the inner layer of coating near the substrate, while R_2_ gives information concerning the resistance of the pore. CPE_3_ and R_3_ describe double-layer capacitance and charge transfer resistance, respectively. This model could fit systems with an outer layer thicker than the deepest one, characterized by few pores. This morphology creates resistances related to mass transport to diffusive phenomena [118,119]. This assumption may be plausible based on SEM images, where inside circular macropores, there was found a compact coating in contact with the substrate. With regard to the bare steel, a simpler R_s_(CPE_3_R_3_) circuit was used, which takes into account the resistance of the solution, the double-layer capacitance, and the charge transfer resistance. The equivalent circuit fits the system well with a χ^2^ value of the order of 10^−4^. The relative error of each parameter is less than 10%. During aging, the values change, and this is attributable to the continuous evolution of the coating in line with the above results. The values showed an increase in overall impedance during the 3-week observation.

### 3.5. ICP-OES Analysis

A further confirmation of the protective effect of the coating was the quantification of metal ions released (Fe, Ni, Cr) in the SBF solution after 21 days of aging. This quantification was carried out by ICP-OES. As reported in Table 3, the concentration of metal ions is very low, below the thresholds dangerous to human health [120]. In addition, in line with our previous work [68], it can be noted that the concentration of calcium and phosphorus ions changes in SBF after aging. This is due to the dissolution and reprecipitation of CaP compounds in SBF [90,91] and, thus, is a further confirmation of the results discussed above.

### 3.6. Cytotoxicity

**Figure 8:**
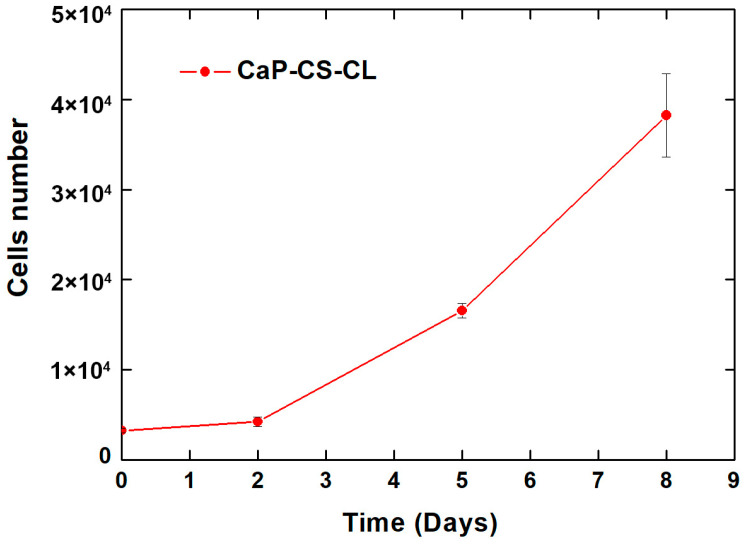
Viability, measured through AlamarBlue reagent, of MC3T3-E1 cells grown in CaP/CS/CL coating treated media.

With regard to the cytotoxicity, an in vitro cellular test was carried out. The CaP/CS/CL sample was soaked in a standard medium for 24 h at an established volume-to-surface ratio according to the ISO standard [121]. MC3T3-E1 pre-osteoblastic cells were maintained in culture for seven days with this treated medium. The cellular growth curve is shown in Figure 8.

From the graphs, it can be appreciated that the number of cells increases in the time from 3 × 10^3^ (seeding days) to approximately 4 × 10^5^ (8 days). These data indicate that a physiological in vitro growth occurred. Therefore, the in vitro cell cytotoxicity assay revealed the non-cytotoxicity—and consequently the biocompatibility—of the CaP/CS/CL coated samples.

## 4. Conclusions

In this work, CaP/CS/CL composite coatings were fabricated on AISI 304 via galvanic deposition. The galvanic coupling between the working electrode and the sacrificial permits the co-deposition of CaP and biopolymers creating a composite coating. SEM images show that the metallic substrate of AISI 304 was thoroughly covered by the deposit. X-ray diffraction reveals that the coating was a mixture of calcium phosphate compounds, in particular brushite and hydroxyapatite, attested by their characteristic diffraction peaks. Nevertheless, only the hydroxyapatite was found at the end of 3-week period of aging in the simulated body fluid due to the total conversion of brushite into hydroxyapatite. The presence of biopolymers was revealed by RAMAN spectroscopy and FT-IR/ATR. Corrosion tests were executed for an aging period of 21 days in simulated body fluid at 37 °C. From these tests, it was observed that the corrosion potential moved toward higher values with respect to uncoated steel. Contemporaneously, corrosion current density decreased, leading to a slow rate of corrosion of the substrate. In line with these results, EIS attests an increase of approximately one order of magnitude in charge transport resistance. A further confirmation of the protective action of coating came from the ICP-OES analyses of the SBF solution before aging, where concentration values well below the threshold limit value were found. In addition, the cytotoxicity test, carried out with MC3T3-E1 preosteoblastic cells, revealed that the CaP/CS/CL coated samples do not influence the normal cellular growth and can be considered not cytotoxic and, consequently, suitable for biomedical applications. Further tests are underway to evaluate whether the increase in collagen concentration modifies the behavior of the coatings.

## Figures and Tables

**Table 1:** Ca/P and Ca/Fe of the coatings before and after aging in SBF calculated from EDS results. The mean deviation is 1.3%.

	Before Aging		Post Aging	
	Ca/P	Ca/Fe	Ca/P	Ca/Fe
**CaP—5 gL^−1^ CS**	1.25	11.7	1.83	no Fe
**CaP—5 gL^−1^ CS—0.2 gL^−1^ CL**	1.29	35.97	1.81	no Fe

**Table 2:** E_corr_ and i_corr_ of CaP/CS/CL and CaP/CS coatings obtained by extrapolation of Tafel’s curves from Figure 6. For comparison, E_corr_ and i_corr_ of the uncoated substrate were also reported. The mean standard deviation was ± 2%.

	Time (Day)					
	0	1	7	14	21	AISI 304
**CaP—5gL^−1^ CS—0.2 gL^−1^ CL**						
E_corr_ (mV)	24	43	85	92	71	−183
i_corr_ (Acm^−2^)	2.75 × 10^−7^	2.12 × 10^−7^	2.45 × 10^−7^	2.15 × 10^−7^	2.01 × 10^−7^	8.92 × 10^−7^
**CaP—5gL^−1^ CS**						
E_corr_ (mV)	−147	−67	−24	−38	68	−183
i_corr_ (Acm^−2^)	4.86 × 10^−7^	2.35 × 10^−7^	2.13 × 10^−7^	2.09 × 10^−7^	2.12 × 10^−7^	8.92 × 10^−7^

**Table 3:** Concentration of ions in the SBF solution after 3-weeks of aging. For comparison, the concentration of Ca and P ions in the as-prepared SBF solution was reported (SBF measured). The mean standard deviation was 0.7%. (SBF calculated is the expected concentration determined from the quantity of the salts used to prepare the solution).

	Concentration (ppm)				
	Fe	Cr	Ni	Ca	P
**SBF (measured)**	0	0	0	103.75	31.74
**SBF (calculated)**	0	0	0	105	31
**AISI 304**	0.037	0	0	82.22	25.44
**CaP—5 gL^−1^ CS**	0.001	0	0	2	110
**CaP—5 gL^−1^ CS—0.2 gL^−1^ CL**	0.001	0	0	4	117

## Data Availability

Not applicable.

## Supplementary Materials

The following supporting information can be downloaded at: https://www.mdpi.com/article/10.3390/polym14235108/s1, Figure S1: OCP curves of: (a) CaP/CS/CL and (b) CaP/CS coatings; Figure S2: Scheme of the Equivalent Circuit. Table S1: Composition of SBF. Table S2: Fitting parameters of impedance measurements of CaP/CS/CL coatings obtained by galvanic deposition during 3-weeks of aging in SBF solution.
