# Supplementary material for: Behavior of Calcium Phosphate–Chitosan–Collagen Composite Coating on AISI 304 for Orthopedic Applications
Source: Polymers (Basel). 2022 Nov 24;14(23):5108. doi: 10.3390/polym14235108 (PMC9735702; doi:10.3390/polym14235108)
Supplement: Supplementary file 1 [file polymers-14-05108-s001.zip › polymers-2026172-supplementary.pdf]

Behavior of Calcium Phosphate-Chitosan-Collagen composite coating on AISI 304 for orthopedic applications

Claudio Zanca[1], Bernardo Patella[1], Elisa Capuana[1], Francesco Lopresti[1], Valerio Brucato[1], Francesco Carfì Pavia[1,2], Vincenzo La Carrubba[1,3], Rosalinda Inguanta[1*]

[1] Department of Engineering, University of Palermo, RU INSTM, Viale delle Scienze, 90133 Palermo, Italy

[2] Consorzio Universitario di Caltanissetta, Corso Vittorio Emanuele 92, 93100 Caltanissetta, Italy

[3] ATeN Center, University of Palermo, Viale delle Scienze, 90133 Palermo, Italy

* Correspondence: rosalinda.inguanta@unipa.it

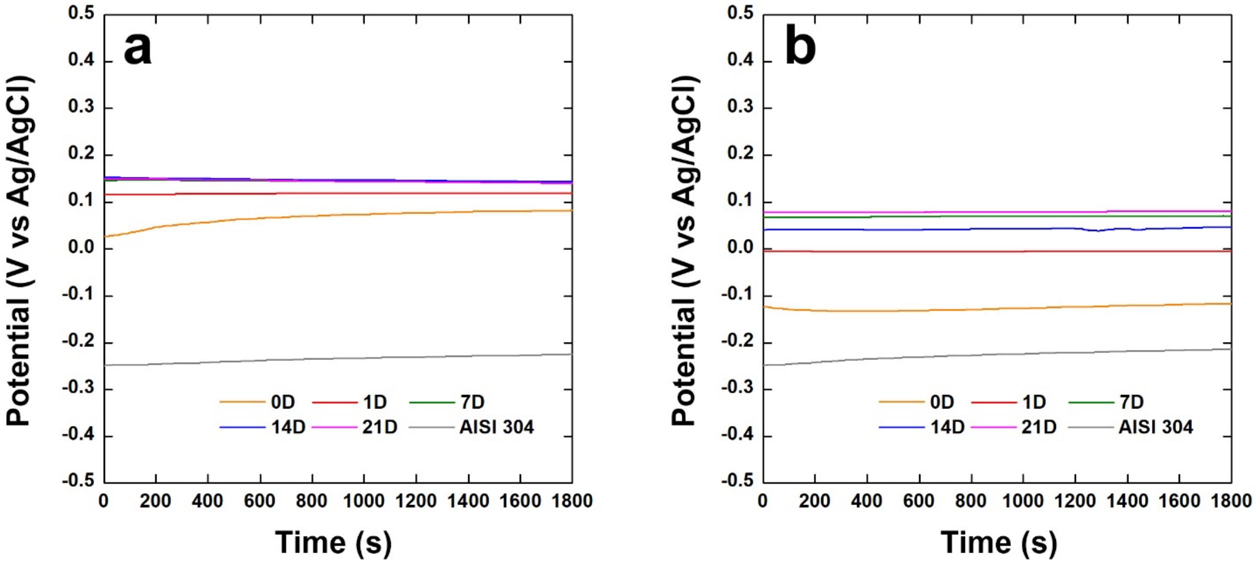

Figure S1: OCP curves of: (a) CaP/CS/CL and (b) CaP/CS coatings.

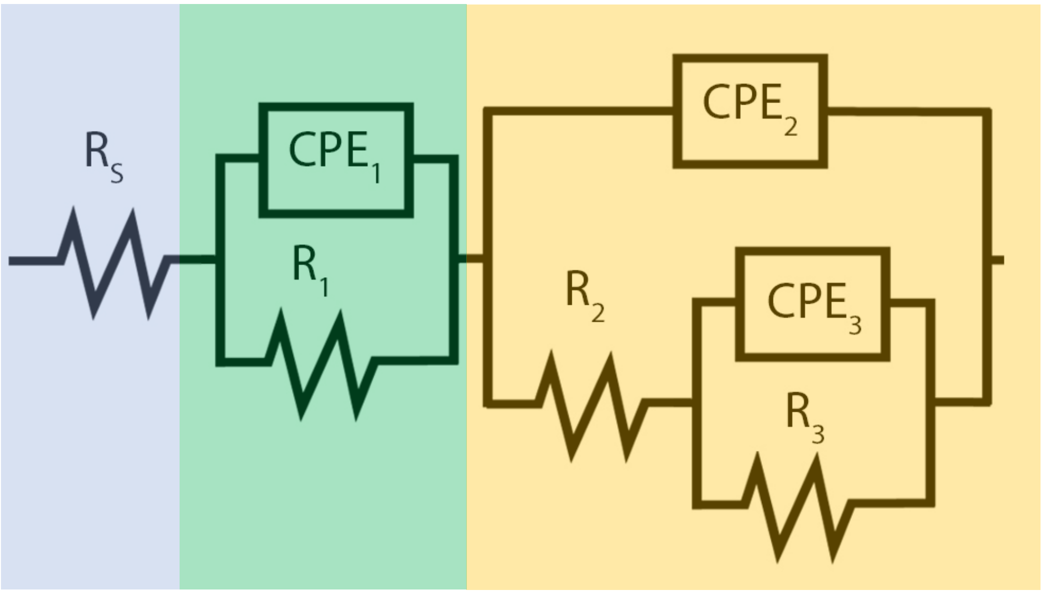

Figure S2: Scheme of the Equivalent Circuit

Reagent	*Composition (g/L)*
**NaCl**	5.403
**$NaHCO_3$**	0.74
**Na_2CO_3**	2.046
**$K_2HPO_4 \cdot 3H_2O$**	0.23
**$MgCl_2 \cdot 6H_2O$**	0.31
**HEPES**	11.93
**$CaCl_2$**	0.29
**Na_2SO_4**	0.072
**1 M NaOH**	0.8 mL

Table S1: Composition of SBF

	**Time (Day)**					
	**0**	**1**	**7**	**14**	**21**	**AISI 304**
	$R_s(CPE_1R_1)(CPE_2(R_2(CPE_3R_3)))$					$R_s(CPE_3R_3)$
**R_s**	52	47.3	23	17	13	36.82
**CPE_1-Y**	7.24E−05	9.36E−05	7.61E−06	3.50E−06	5.67E−06	-
**CPE_1-n**	0.9193	0.975	0.4906	0.524	0.5064	-
**R_1**	190	1010	1166	2738	2046	-
**CPE_2-Y**	2.77E−05	1.22E−05	5.12E−05	1.76E−05	2.59E−05	-
**CPE_2-n**	0.644	0.5325	0.526	0.6553	0.6061	-
**R_2**	6.39E+04	1.80E+05	2.65E+04	4.71E+04	3.25E+04	-
**CPE_3-Y**	4.89E−06	4.96E−05	3.88E−05	3.92E−05	4.12E−05	3.60E−05
**CPE_3-n**	0.5157	0.7521	0.8224	0.6799	0.7055	0.8046
**R_3**	1.12E+04	3.50E+04	1.00E+06	2.62E+05	3.46E+05	3.07E+05

Table S2: Fitting parameters of impedance measurements of CaP/CS/CL coatings obtained by galvanic deposition during 3-weeks of aging in SBF solution.
